# International Sanitation

**Published:** 1901-12

**Authors:** 


### International Sanitation.

International Sanitation is the proposition made by Surgeon-General Wyman to the Pan-American republics, in order to eliminate yellow fever from the seaport cities or towns in which it has so long been endemic. The measures are by sanitary improvements of harbors, sewerage, soil drainage, paving and the elimination of infection from buildings. To bring about reform Dr. Wyman proposes an International Sanitary Commission to consist of five members, no two from the same republic, to be appointed by the Bureau of American Republics; in the commission one shall be a diplomat, one a jurist, one a physician, one a sanitary engineer, one a commercial representative. The commission is to report to the president of the republic in which the city is situated, as to the sanitary measures deemed necessary, and within a year if these have not been begun or contracted for, a tonnage tax or duties are to be imposed upon specified imports by the nations entering into the agreement until the needed sanitary work has been completed. This plan has about it the ring of the genuine metal, and we hope the various nations concerned will recognize their duty and opportunity. As Dr. Wyman earnestly pleads: "The time has come when common action should be taken. Trade and a higher civilization both demand that we should attend to this matter now. Our American republics have an opportunity to inaugurate a new system for the advancement of civilization. Tropical and semi-tropical countries are not necessarily unhealthy. Their apparent unhealthfulness is not due to climate, but to faulty sanitation, or lack of it."—*American Medicine.*

